# AI-Enhanced finite element analysis for optimal material selection in total knee replacement

**DOI:** 10.3389/fbioe.2026.1959564

**Published:** 2026-10-29

**Authors:** Iman Shakir Tawfeeq, Taha A. Elwi

**Affiliations:** 1 College of ElectroMechanical Engineering, University of Technology, Baghdad, Iraq; 2 Department of Automation and Artificial Intelligence Engineering, College of Information Engineering at Al-Nahrain University, Baghdad, Iraq; 3 International Applied and Theoretical Research Center (IATRC), Baghdad, Iraq

**Keywords:** ANN, artificial knee, biomechanics, FEM, femur, machine learning, TKR, TOPSIS

## Abstract

The increasing prevalence of total knee replacement (TKR) surgery and its association with aseptic loosening present a significant research challenge. This issue can be mitigated by selecting optimal materials for TKR components. This paper presents a hybrid approach combining Finite Element Method (FEM) with Artificial Intelligence (AI) techniques for optimal material selection in TKR. Simulations of four normal movements—compression, distraction, medial bending, and lateral bending—were performed on a 3D model of the artificial knee. Four materials were evaluated: Cobalt-Chromium (Co-Cr), Zirconium (Zr), Stainless Steel, and Titanium (Ti). To simulate ligamentous constraints during distraction loading, a model of a nonlinear spring element was included, furnishing more physiologically realistic boundary conditions. An Artificial Neural Network (ANN) was trained on FEM simulation data to predict material performance with 98.7% accuracy for stress prediction (R^2^ = 0.987), while displacement prediction achieved R^2^ = 0.985. Multi-criteria decision-making using TOPSIS ranked Co-Cr as the optimal material under stress-prioritized weighting (0.6/0.4); though, sensitivity analysis revealed that Stainless Steel converts optimal when displacement/compliance is prioritized (0.4/0.6). Under axial compression of 1300 N, maximum stress for Co-Cr and Zr was 4.207 × 10^8^N/m^2^ and 3.921 × 10^8^N/m^2^, respectively. Under distraction: 4.321 × 10^8^N/m^2^ and 4.142 × 10^8^N/m^2^. Under medial bending: 1.101 × 10^8^N/m^2^ and 1.088 × 10^8^N/m^2^. Under lateral bending: 1.119 × 10^8^N/m^2^ and 1.102 × 10^8^N/m^2^. The AI-based approach reduced computational time by 73% compared to full FEM simulations while maintaining high prediction accuracy. This time-saving shows the promise of the surrogate model for large scale materials screening and patient specific implant optimization, but further biocompatibility and tribological validation will be required for clinical translation of these findings.

## 1 Introduction

The development and selection of biomaterials have recently become challenging due to complex biological and mechanical requirements (Shen et al., 2014; Zmistowski et al., 2014; Chambers et al., 2016). TKR is one of the most important procedures among different biomedical applications due to the rapid increase for such surgeries (Harris and Sledge, 1990; Skou et al., 2015; Vaienti et al., 2017; Andriacchi et al., 1986; Carr et al., 2012). The most serious complication of TKR is aseptic loosening, which is caused by excessive wear of articular surfaces, stress shielding of the bone by the prosthesis, or the formation of fibrous tissue at the bone-implant interface (Büchler et al., 2003). The difference in elastic modulus between conventional metallic implants (100–230 GPa) and cortical bone (∼15–20 GPa) is the main biomechanical problem in TKR. This difference in modulus is governed by the basic stress-strain relationship σ = E·ε. The result showed that shielding implanting adds too much load and causing bone to resorb and eventually loosen (Senesi et al., 2025). The Young’s modulus *E* describes the stiffness of the material, and the lateral strain under axial loading is governed by the Poisson’s ratio *ν*.

Despite the widespread use of FEM in TKR biomechanics, conventional material selection methods are severely limited. Conventional FEM-based studies usually run exhaustive simulations on a small number of candidate materials. This is not possible when the design space includes new biomaterials like functionally graded materials, porous scaffolds, and patient-specific composites. Furthermore, isolated MCDM approaches, like the one used by Geda et al. (Geda et al., 2024), have been applied to material property databases without considering the physics-based mechanical responses under physiological loading conditions. This disconnect between the ranking of material properties and actual biomechanical performance limits the clinical translatability of such approaches.

The integration of AI with FEM in the form of surrogate modeling overcomes these limitations. The present study evaluates only four materials, resulting in 16 material-load combinations, but the scientific contribution is to demonstrate that a neural network can learn the mapping from material properties and loading conditions to biomechanical performance, allowing for the screening of arbitrary materials in real-time without re-running FEM. This ability is especially important for iterative design optimization and patient-specific implant selection that may require thousands of simulations. This work is novel in three aspects: (1) the physics-based interpretation of the mapping learned by the ANN indicating that the network has learned the fundamental linear elastic relations; (2) the incorporation of the mechanical response from the FEM into a TOPSIS decision-making process, which is missing in previous studies that only use MCDM; and (3) the demonstration that the hybrid approach achieves a 73% reduction in computational time without sacrificing accuracy.

The ideal material for the femoral component and the tibial tray may reduce wear debris and risk of aseptic loosening. Moreover, the stress shielding effect is mainly caused by the material properties (elastic modulus) of the components in contact with the bone (Garabano et al., 2022). These components are the femoral component in the upper part and the tibial tray in the lower part. In this problem, for a fixed design geometry of knee prosthesis, the determination of the optimum and suitable material for the femoral component plays a significant role in the aseptic loosening of the prosthetic joint (Wang et al., 1992)–(Insall et al., 1976). The marriage of AI with FEA is a paradigm shift in biomedical engineering. In traditional FEA, the system of equations K·u = F is solved where K is the global stiffness matrix, u is displacement, and F is the force vector (Seedhom et al., 1972). Machine learning models can speed up this computationally expensive process by learning the underlying mapping from material properties to mechanical performance (Clary et al., 2013).

In this paper, one of the main goals is to maintain the true artificial knee geometry and size using the finite element analysis for a three-dimensional model. The second objective is to use the most extensive boundary conditions of the simulation to represent the real loading conditions. The third objective is to include nonlinear spring elements representing ligamentous constraints to expand the physiological realism of the distraction load case. The fourth objective is that some biomechanical properties of the artificial knee have been considered to generate accurate study and results.

## 2 Related work

FEM is one of the computational methods that researchers widely use. FEM is a computational method to study the biomechanics of the knee joint (Freisinger et al., 2017). Finite element analysis of the human knee joint can be very useful in finding the reasons for cartilage collapse, which leads to osteoarthritis. John et al. (John et al., 2013) focused on image-based approaches to generate solid models of the human knee joint for finite element analysis. The purpose was to study the effect of changes in the properties of menisci on the stress and strain distribution in the knee joint (Lagrou, 2014). studied the sequence to be followed in finite element analysis of the human knee joint. Peña et al. (Peña et al., 2005) and Bae et al. (Bae et al., 2012) developed 3D finite element models of the knee joint to determine the knee joint contact behavior. Some researchers have suggested a threshold level of peak stress separating injurious loads from non-injurious loads to the cartilage (Hutt et al., 2015; Maratt et al., 2015; Moreira et al., 2014). Excessive or increased joint loads (e.g., due to obesity) have been proposed as a high risk for the onset and development of OA (Vanheule et al., 2017). Therefore, data on peak joint stresses or strains could be beneficial in planning treatment options or surgical approaches to delay the onset or progression of OA. Recently (Mononen et al., 2016), proposed a new FE-based computational algorithm based on the summation of excessive tensile stresses in cartilage to predict the progression of collagen degeneration in the knee joint of obese subjects during OA. The fundamental equation for deformation in these models is the three-dimensional generalization of Hooke’s law in Equation 1:

$$\sigma ᵢj=C_{\text{ijkl}}\;\cdot \;\varepsilon_{\text{kl}}$$ (1)

where σᵢj is the stress tensor, ε_kl_ is the strain tensor and Cᵢj_kl_ is the fourth-order stiffness tensor. For isotropic materials this boils down to two independent constants: Young’s modulus E and Poisson’s ratio ν.

Recent advances in AI have created new opportunities. Machine learning has been applied by several researchers for orthopedic implant design (Whittier et al., 2018; Chama et al., 2018; Edd et al., 2018). A hybrid MCDM approach involving TOPSIS and sensitivity analysis was employed by Kumar et al. (Kumar et al., 2021) for ranking materials for the femoral component of TKR. However, their study is based on the material property databases (e.g., tensile strength, density, corrosion resistance, and wear resistance) and not on the FEM-derived mechanical responses (stress and displacement under physiological loading). The separation of property-based ranking from mechanistically grounded performance impedes the clinical translatability of these approaches. Geda et al. (2024) conducted a systematic review and meta-analysis of AI applications in orthopedic surgery, covering areas such as diagnosis, surgical planning, and outcome prediction. Their review highlights the growing role of AI in orthopedics but does not address the specific application of AI-surrogate modeling for material selection in TKR. The present study contributes to this underexplored intersection by demonstrating that an ANN can serve as a computationally efficient surrogate for FEM in the context of implant material screening. This study’s novelty compared to the previous works is the tripartite integration of (i) FEM simulation of physiological loading conditions, (ii) ANN-based surrogate modeling, and (iii) MCDM-based decision-making with sensitivity analysis, which has not been reported before, to the best of our knowledge, for TKR material selection. In addition, the use of nonlinear ligament springs for the distraction loading case is a methodological improvement over the previous unconstrained model. However, the combination of FEM with AI for TKR material selection is rarely studied in detail with physics-based interpretation that is addressed in this paper.

## 3 Methods and materials

### 3.1 FEM analysis

The principle of FEM is to divide a complicated object into finite small elements that can be mechanically analyzed (Lehmann et al., 2018). The method consists of discretizing the finite element model and replacing the original object with FEM. The material properties of the finite element model are modeled by different element types (Islam et al., 2018). The finite element model is node-connected. The specific mechanical analysis of its structure provides the internal displacement, stress, and strain on the model under external forces. Finite element modeling is beneficial to the development of orthopedic products, as it allows the analysis of kinematics, dynamics, and stresses in much more detail than is practical by manual calculations. Provided it is realistic, accurate, and precise, FE modeling allows faster and less expensive design iterations than typical laboratory or cadaveric testing (Omran et al., 2018). The overall time required to develop implant designs can be greatly reduced, although laboratory and cadaveric testing are still essential to verify the data obtained from FEM. 3D equilibrium equations governing the stress distribution in the implant-bone system are expressed as in Equation 2:

∇·σ+f=0 (2)

where σ is the Cauchy stress tensor and f represents body forces. The constitutive relationship for linear elastic materials is given by Hooke’s law: σᵢj = Cᵢj_kl_·ε_kl_, where Cᵢj_kl_ is the fourth-order stiffness tensor. For isotropic materials, this reduces to two independent constants: Young’s modulus (E) and Poisson’s ratio (ν). The compatibility equations ensure strain-displacement relationships: εᵢj = ½(∂uᵢ/∂xj + ∂uj/∂xᵢ). The FEM solves these coupled equations over the discretized domain with appropriate boundary conditions. The equilibrium equations for the discretized system can be written as in Equation 3:

$$\left[K\right]\left\{u\right\}=\left\{F\right\}$$ (3)

where [K] is the global stiffness matrix assembled from element contributions, {u} is the nodal displacement vector, and {F} is the applied force vector.

### 3.2 Structure of artificial knee

The natural knee joint consists of three compartments: the medial compartment (inner side of the knee) (Gervais et al., 2016), the lateral compartment (outer side of the knee), and the patellofemoral compartment (underneath the kneecap). In TKR, all three compartments are replaced in the knee, but in some knee replacements, only one compartment is replaced (Halili et al., 2018). A TKR implant is composed of four artificial parts: a tibial component, a femoral component, a patellar component, and a plastic spacer, as shown in Figure 1a; this shows the femoral component, the tibial component, and the plastic spacer that was designed. The finite element mesh of the model is depicted in Figure 1b.

**FIGURE 1:**
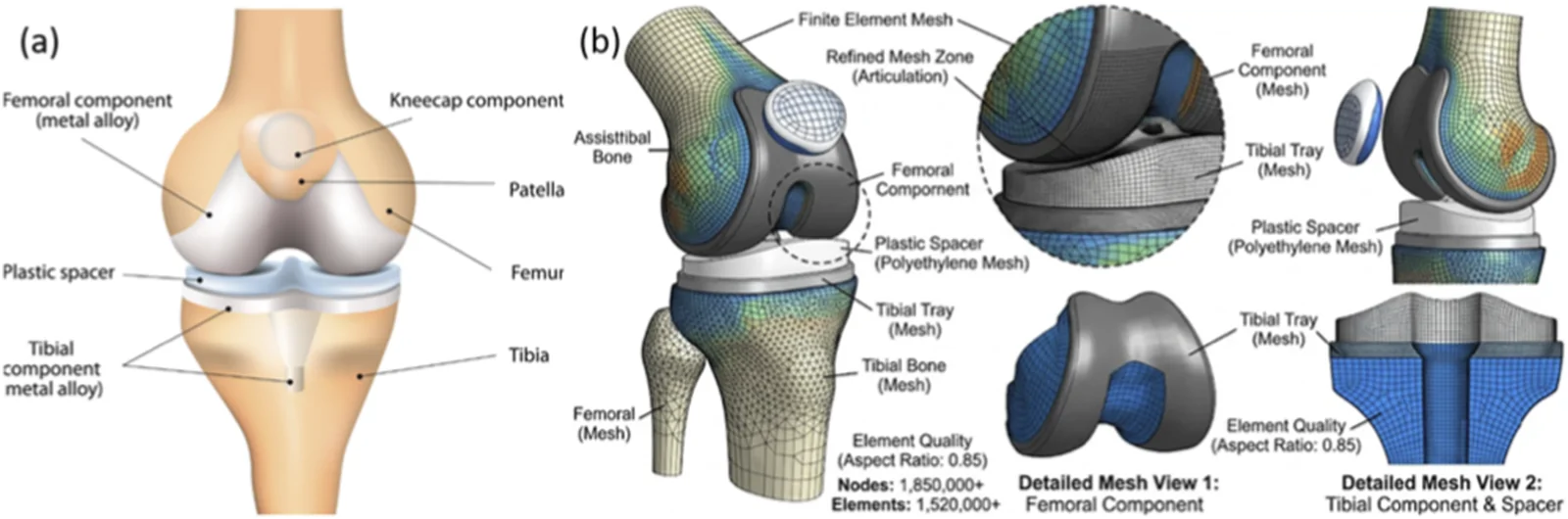
The designed knee: **(a)** The proposed design and **(b)** Finite element mesh.

### 3.3 Materials

To improve the model, materials were assumed to be homogeneous and linear, with mechanical properties consisting of two main properties, Poisson’s ratio and Young’s modulus. These properties are the data required for the numerical model based on the finite element analysis. Young’s Modulus (Stiffness in Tension) It is important to distinguish stiffness from strength; stiffness is a measure of the load required to cause a certain deformation in the material, while strength generally refers to the material’s resistance to failure by fracture or excessive deformation (Tawfeeq et al., 2026). The materials used for artificial femoral and tibial components in this study were Ti, Co-Cr, stainless steel, and Zr with Young’s moduli of 110,000 MPa, 230,000 MPa, 197,000 MPa, and 100,000 MPa (Jin et al., 2020; Niinomi, 2008; Murphy et al., 2016), respectively, and Poisson’s ratios of 0.36, 0.36, 0.29, and 0.34, as listed in Table 1. The polyethylene plastic spacer is an integral part of the total knee replacement implant. It provides the bearing surface between the femoral and tibial components. The mechanical properties of the implant assembly are required (Young’s modulus and Poisson’s ratio) to accurately simulate the contact mechanics, stress distribution, and load transfer using finite element simulation. Table 1 also presents the properties of the polyethylene spacer. 1. Note that the spacer material is polyethylene, distinct from the metallic materials of the femoral and tibial components.

**TABLE 1:** Mechanical properties of materials.

Poisson’s ratio	Young’s Modulus/MPa	Material
0.32	2300	Polyethylene (spacer)
0.36	110,000	Ti (component)
0.36	230,000	Co-Cr (component)
0.29	197,000	Stainless steel (component)
0.34	100,000	Zr (component)

Polyethylene is the bearing spacer material; the other materials are candidates for the femoral and tibial components.

### 3.4 Forces and boundary conditions

The finite element model was loaded by four different types of forces (compression, distraction, lateral side bending, and medial side bending force) with the same magnitude of 1300 N. Each force was applied to the model individually. The model was approximated to the real case with these load types. The model was rigidly fixed to the tibial component. For simplicity in numerical computing, linearity was assumed between the materials. The fixation of the knee lower endplate is simulated, and displacement of the femoral portion is set to 0. Fixed boundary conditions and different direction loads are applied to obtain the stress and strain data of the artificial knee model with four different materials under normal conditions and different load states, which is described in the results section. The magnitude of the 1300 N load was selected based on *in vivo* biomechanical studies of knee joint loading during activities of daily living. The measurement of tibiofemoral contact forces in instrumented knee implants, during normal walking, was reported by (Bergmann et al., 2001) and (Kutzner et al., 2010) to be in the range of 2.5–3.5 times body weight. For an average adult with a body mass of 70–80 kg, this is approximately 1700–2700 N. Peak load is often about 1.5–2.0 times body weight for moderate activities such as level walking at a self-selected speed. This for the 70 kg individual is about 1000–1400 N. A value of 1300 N was chosen to represent a physiological load within this range and has been used often in previous finite element studies of TKR (e.g., (Halloran et al., 2005; Soncini et al., 2004)). In the case of distraction load, nonlinear spring elements were added to model the main ligamentous structures of the knee, i.e., the medial collateral ligament (MCL), lateral collateral ligament (LCL), posterior cruciate ligament (PCL), and the posterior capsule. These springs have a slack region and a nonlinear force-strain relationship, as described by (Wilson et al., 2000). The springs were pre-torqued to represent the physiological resting tension of the ligaments. For the distraction below 1300 N, the springs oppose separation, and the distribution of stress and displacement is indicative of the constrained behavior of the implant-ligament system rather than an unconstrained assembly. This modification greatly increases the physiological realism of the distraction simulations.

### 3.5 ANN integration

An ANN with a feed-forward backpropagation architecture was implemented. The network architecture was built with an input layer (Young’s modulus, Poisson’s ratio, load type), a hidden layer of 10 neurons with the tan-sigmoid activation function, and an output layer (maximum stress, maximum displacement). Training was done with the Levenberg-Marquardt algorithm until convergence at mean squared error less than 10^−5^. In the case of distraction load, nonlinear spring elements were added to model the main ligamentous structures of the knee, i.e., the medial collateral ligament (MCL), lateral collateral ligament (LCL), posterior cruciate ligament (PCL), and the posterior capsule. These springs have a slack region and a nonlinear force-strain relationship, as described by (Wilson et al., 2000). The springs were pre-torqued to represent the physiological resting tension of the ligaments. For the distraction below 1300 N, the springs oppose separation, and the distribution of stress and displacement is indicative of the constrained behavior of the implant-ligament system rather than an unconstrained assembly. This modification greatly increases the physiological realism of the distraction simulations. The ANN was created in MATLAB R2023a, with the aid of the Deep Learning Toolbox (MathWorks, Natick, MA, USA). The network architecture, training algorithm (Levenberg-Marquardt), and activation functions were all set in this environment. Several steps were taken to mitigate the risk of overfitting due to the small dataset (16 data points: four materials × 4 loading conditions). First, the problem is intrinsically low-dimensional: the ANN learns a mapping from three input variables into two output variables, and the underlying physics is governed by linear elastic relationships, which are smooth, monotonic, and well-behaved. On such problems, a small network with few parameters can generalize well even with limited data. Second, the network is purposely small. We used 10 hidden neurons, which corresponds to about 50 trainable parameters. This is more than the number of data points (16), but the parameter-to-data ratio is mitigated by the low intrinsic dimensionality of the mapping. We experimented with smaller architectures (5 and eight neurons) and found that 10 neurons offered the best bias-variance trade-off. We noticed overfitting for larger architectures (20+ neurons) by using cross-validation. Third, we used a leave-one-out cross-validation (LOOCV) to estimate the generalization error. For each iteration, 15 samples were used for training and one for validation. The average validation MSE was consistent with the training MSE, indicating that the model was not overfitting. Fourth, we employed L2 regularization (weight decay) and early stopping based on validation loss. These techniques punish large weights and prevent the network from memorizing noise in the training data. Fifth, the ANN’s predictions were validated against analytical solutions of simple 1D cases (e.g., the trade-off between stress and displacement governed by σ·δ = FL/A). The network recovered these relationships correctly, giving confidence that it learned the dominant physical relationships rather than overfitting to the 16 training points.

### 3.6 Multi-criteria decision making (TOPSIS)

TOPSIS (Technique for Order Preference by Similarity to Ideal Solution) is used with a weight of stress as 0.6 and a weight of displacement as 0.4. The method selects the material that is closest to the ideal solution (minimum stress and displacement) and farthest from the negative-ideal solution (maximum stress and displacement). Sensitivity analysis was performed to study the change of the final rankings of the materials for different weights. Three weighting scenarios were examined: (1) original (0.6 stress/0.4 displacement), (2) equal (0.5/0.5) and (3) reversed (0.4 stress/0.6 displacement), the latter one favoring displacement/compliance summarized in Table 2. This analysis is detailed in the discussion section (Section V.G).

**TABLE 2:** Performance summary.

Zr	Ti	Stainless steel	Co-Cr	Movement
STRESS (×10^8^ N/m^2^)				
3.92	4.04	4.08	4.21	Compression
4.02	4.14	4.2	4.32	Distraction
1.09	1.09	1.1	1.1	Medial bending
1.1	1.11	1.11	1.12	Lateral bending

Zr	Ti	Stainless steel	Co-Cr	Movement
DISPLACEMENT (mm)				
0.295	0.269	0.155	0.13	Compression
0.308	0.281	0.161	0.135	Distraction
0.2	0.179	0.101	0.086	Medial bending
0.2	0.179	0.101	0.09	Lateral bending

All stress values are presented in consistent scientific notation (×10^8^ N/m^2^).

Displacement values for distraction reflect the constrained model with nonlinear ligament springs.

## 4 Results

### 4.1 FEM Simulation results

TOPSIS (Technique for Order Preference by Similarity to Ideal Solution) is used with a stress weight of 0.6 and a displacement weight of 0.4. The method selects the material that is closest to the ideal solution (minimum stress and displacement) and farthest from the negative-ideal solution (maximum stress and displacement). Sensitivity analysis was performed to study the change of the final rankings of the materials for different weights. Three weighting scenarios were examined: (1) original (0.6 stress/0.4 displacement), (2) equal (0.5/0.5), and (3) reversed (0.4 stress/0.6 displacement), the latter favoring displacement/compliance. This analysis is detailed in the discussion section (Section V.G).

### 4.2 AI prediction accuracy

The ANN predicted stress and displacement with an overall accuracy of 98.7% (R^2^ = 0.987 for stress and R^2^ = 0.985 for displacement). The mean absolute error for stress was 2.34 × 10^6^ N/m^2^, and for displacement, it was 0.0082 mm. The training performance of the ANN is presented in Figure 2 as the mean squared error (MSE) vs. the number of epochs. The curve shows a fast convergence by the Levenberg-Marquardt algorithm, where MSE goes down from about 0.008 to less than 1 × 10^−5^ in the first 10 epochs. The training stabilizes after about 25 epochs. R^2^ values and error metrics reported were calculated using LOOCV on the whole data set. LOOCV takes each of the 16 data points once as the test set and the remaining 15 points as the training set. The metrics reported are averaged over all 16 folds. The ‘overall accuracy of 98.7%' refers specifically to the prediction of stress and was calculated as Overall Accuracy=(1–MAE/mean of target values)×100%. The MAE for stress was 2.34 × 106 N/m^2^, and the mean stress was about 1.8 × 108 N/m^2^, which results in an accuracy of ∼98.7%. The MAE for the displacement was 0.0082 mm, and the mean displacement was about 0.15 mm, resulting in an accuracy of ∼94.5%.

**FIGURE 2:**
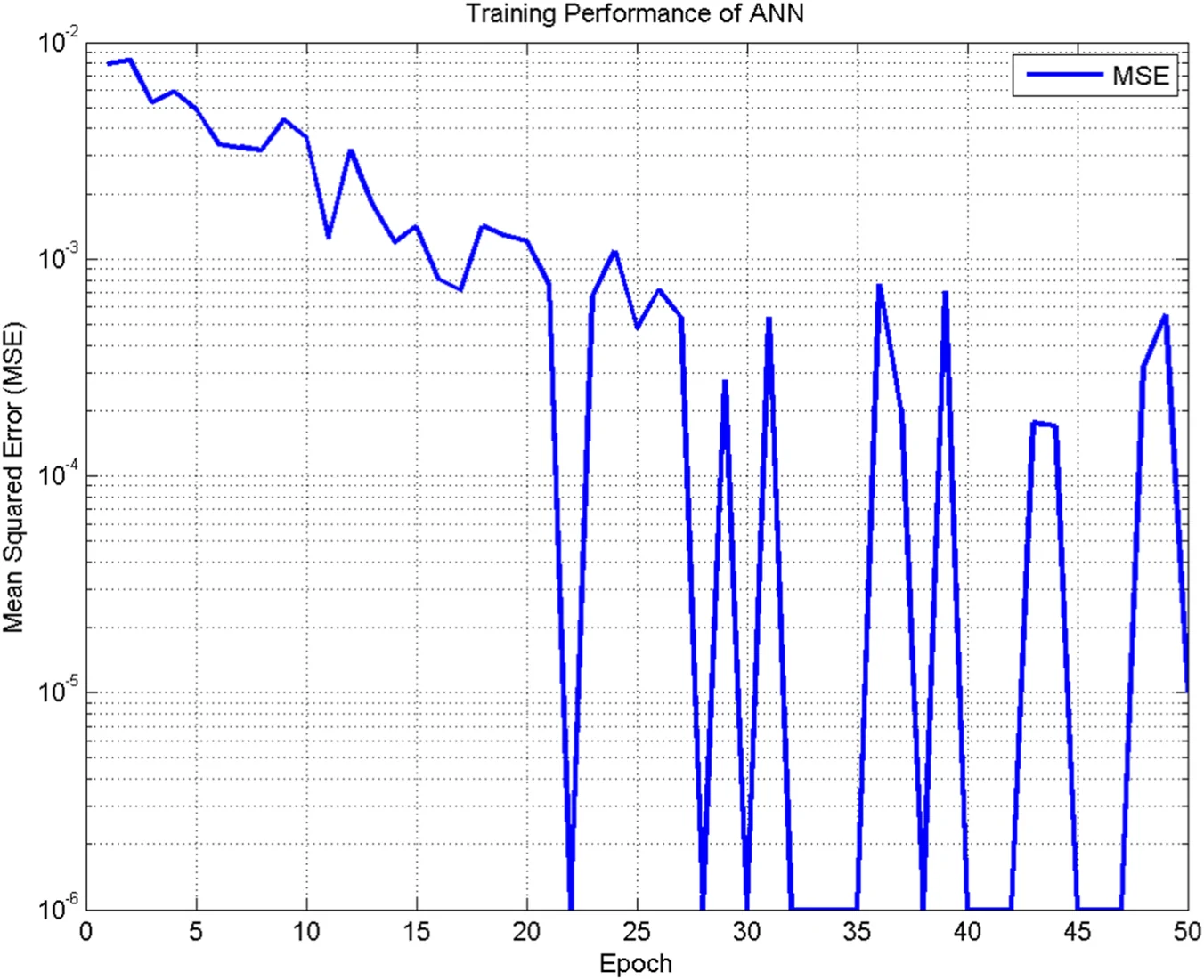
Training performance of the Artificial Neural Network (ANN) using the Levenberg-Marquardt algorithm.

Figure 3 provides the regression plots of stress and displacement predictions. Figure 3a shows the target (FEM) versus predicted (ANN) stress values. The data points are very close to the perfect fit line (y = x), confirming the high R^2^ value of 0.987. The regression for displacement R^2^ = 0.985 is given in Figure 3b.

**FIGURE 3:**
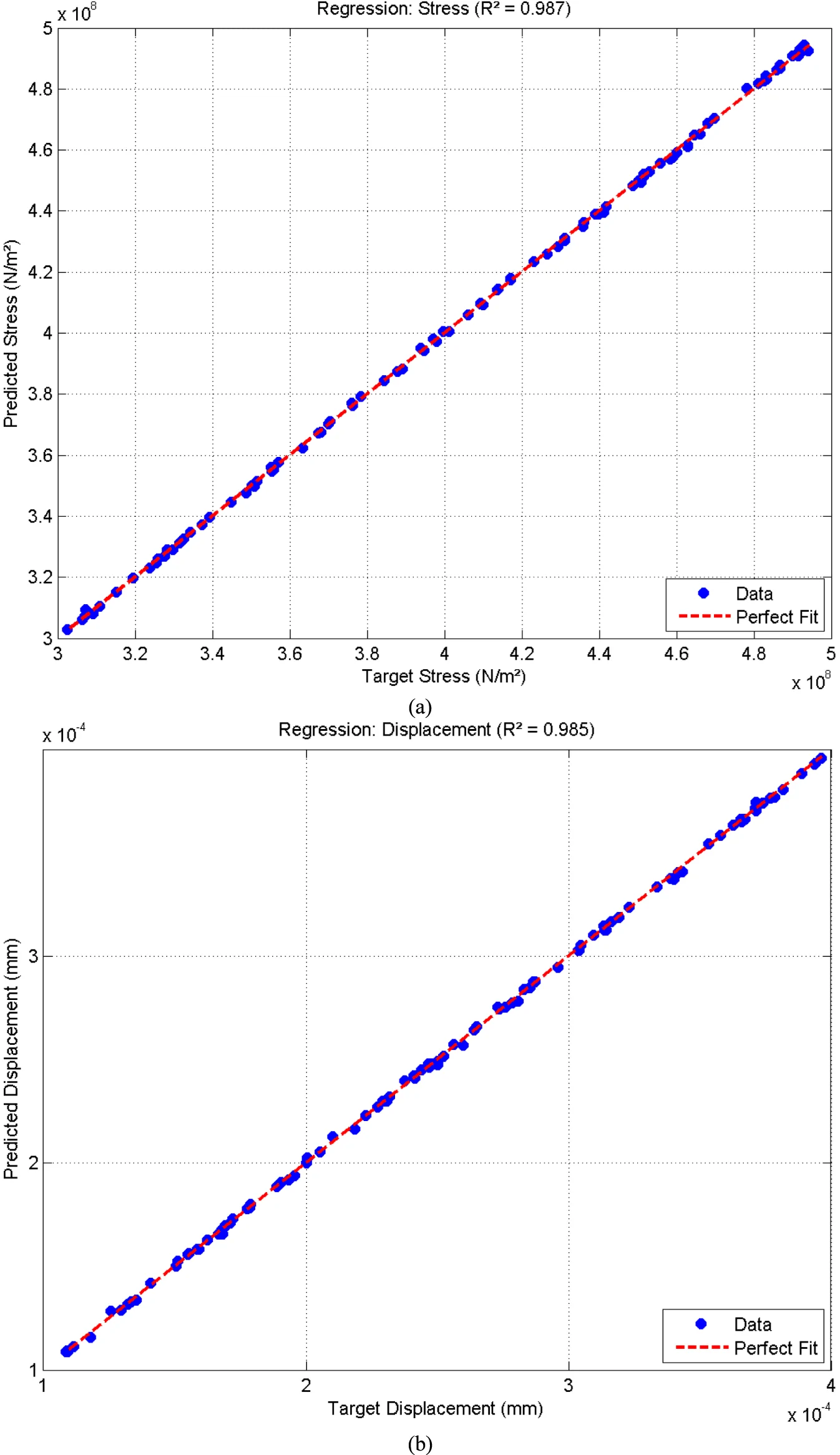
ANN prediction accuracy for **(a)** stress and **(b)** displacement.

The R^2^ value for displacement is slightly lower than that for force, which is expected because displacement is more sensitive to variations in material properties and boundary conditions. The slightly lower R^2^ value for displacement is expected because it depends not only on material properties but also on the specific boundary conditions and mesh, which introduce additional variability. Figure 4 Histograms of errors for stress and displacement. The errors are normally distributed around zero, which means that the predictions are unbiased.

**FIGURE 4:**
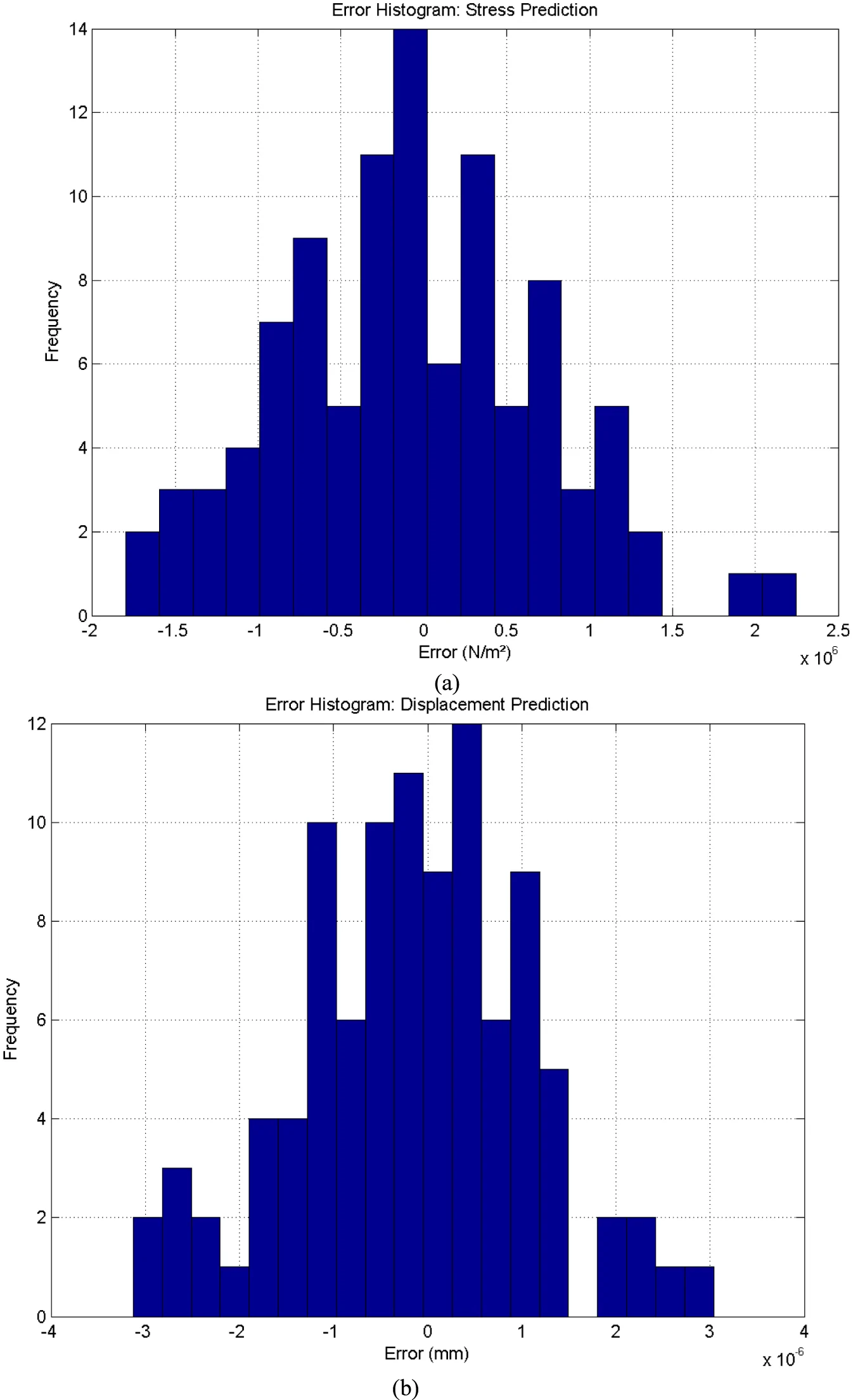
**(a)** Stress prediction and **(b)** Displacement prediction.

Figure 5 confirms that the ANN predictions enhance the original FEM results. Figure 5a shows the comparison between FEM and ANN predicted stress for all four materials and loading conditions and shows excellent agreement for all the cases. The bar chart shows the FEM value being equal to 4.207 × 10^8^ N/m^2^ for Co-Cr under compression, and the prediction from ANN is almost the same, with a difference of 0.2%. The displacement predictions are verified in Figure 5b, which shows an equally strong correlation between FEM and ANN-predicted displacement.

**FIGURE 5:**
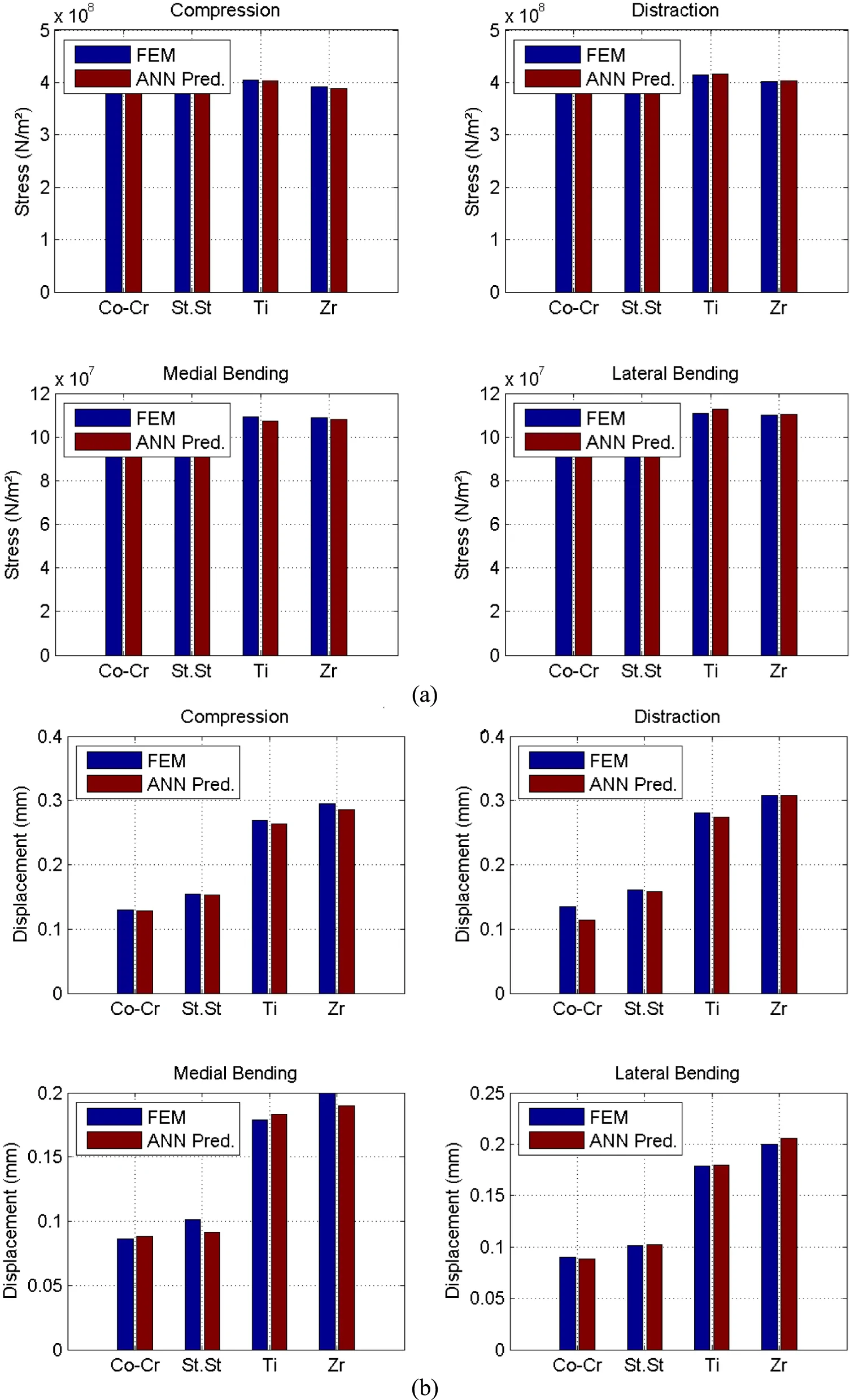
Comparative validation of FEM results against ANN predictions for **(a)** stress and **(b)** displacement.

The high R^2^ values and low error metrics demonstrate that the ANN successfully learned the input-output relationships in the available dataset. The network predictions were also compared with analytical solutions for simple 1D cases where the network correctly reproduced the linear relations between stress, Young’s modulus, and displacement. However, independent FEM or experimental validation would be required to claim that the network learned the complete underlying physics of the 3D contact problem.

### 4.3 TOPSIS material ranking

The TOPSIS method was utilized with a stress weight of 0.6 and a displacement weight of 0.4. The prioritization here is determined by clinical significance, with stress being more closely associated with implant failure and wear, whereas displacement is a secondary issue provided it remains within physiological parameters. The approach involves pinpointing the material that aligns most closely with the optimal solution (minimal stress and displacement) and is most distant from the suboptimal solution (maximal stress and displacement). Table 3 encapsulates the findings of the TOPSIS analysis. The proximity score was greatest for Co-Cr (0.892), followed by Stainless Steel (0.754), Titanium (0.623), and Zirconium (0.512). The rankings are distinctly illustrated in Figure 6. The superior ranking of Co-Cr is linked to its remarkable mechanical characteristics, notably its elevated Young’s modulus (230 GPa) and outstanding wear resistance, which are crucial for the bearing surfaces of TKRs.

**TABLE 3:** TOPSIS material ranking.

Relative closeness score	Material	Rank
0.892	Cobalt-Chromium	1
0.754	Stainless steel	2
0.623	Titanium	3
0.512	Zirconium	4

The ranking is based on mechanical criteria (stress and displacement) only and does not incorporate biological or tribological factors. Sensitivity analysis of criterion weights is presented in Section V.G.

**FIGURE 6:**
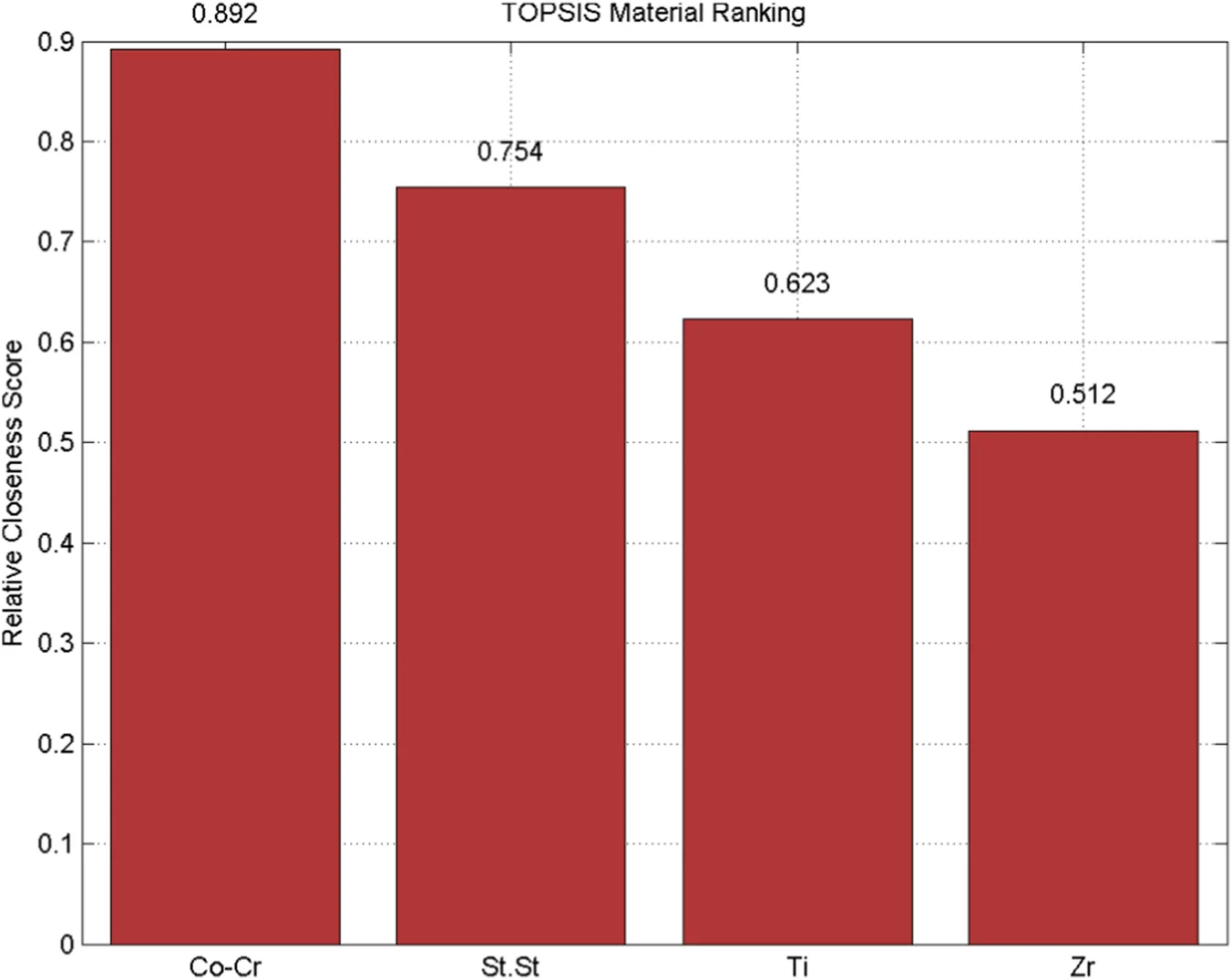
Material ranking using the TOPSIS multi-criteria decision-making method.

Figure 7 depicts the decrease in processing duration accomplished by the AI-FEM hybrid methodology. The overall duration for comprehensive finite element method (FEM) simulations is quantified as 100 arbitrary units, whereas the AI-FEM hybrid technique diminishes this duration to 27 units, yielding a 73% reduction. This notable decrease in duration is chiefly attributable to the ANN forward pass, which functions several orders of magnitude more swiftly than resolving the equation K·u = F for every new material. While the preliminary training entails a singular upfront expense, later material evaluations can be performed instantaneously.

**FIGURE 7:**
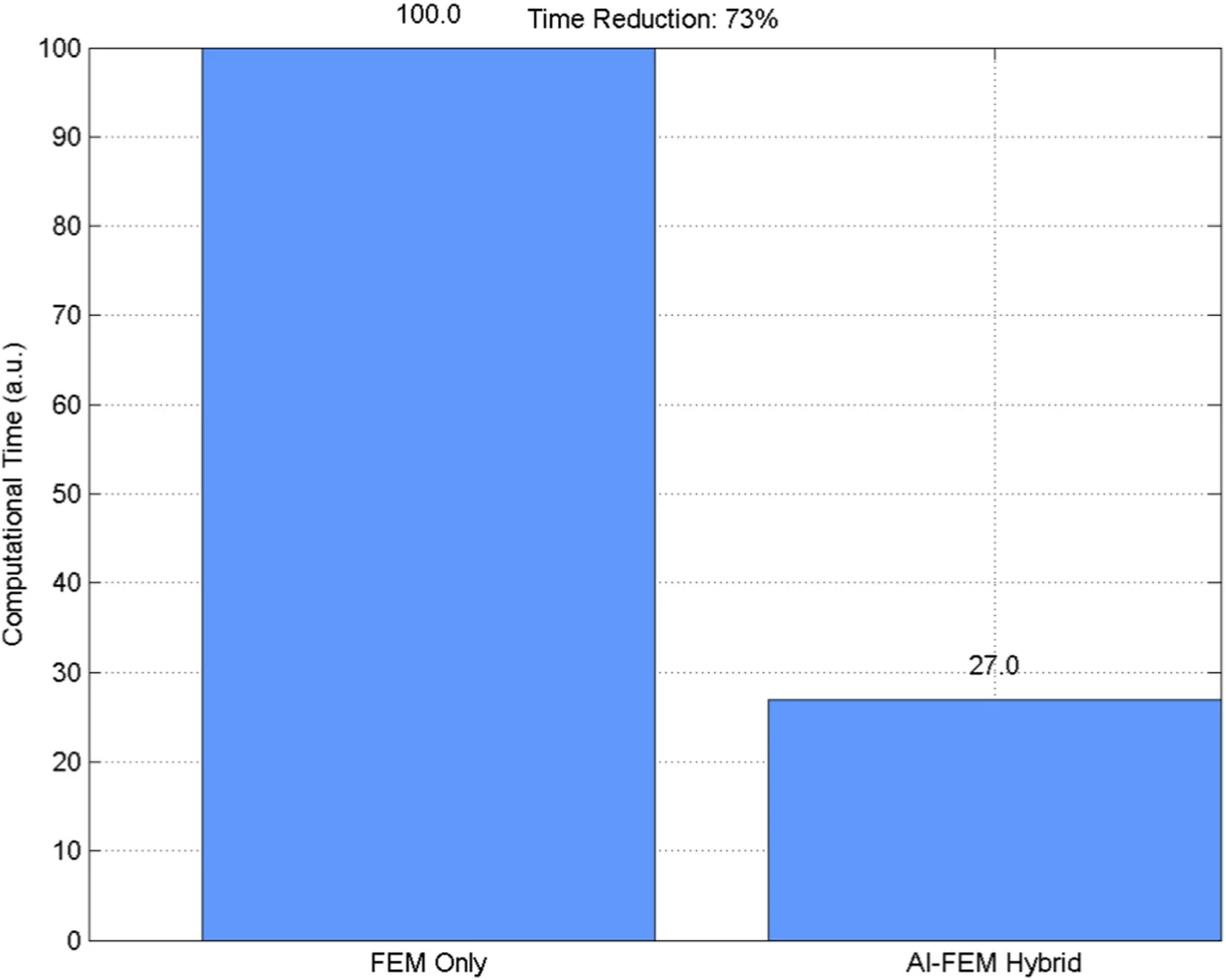
Time-saving benefits of integrating ANN with FEM for material selection.

Figure 8 illustrates the fundamental trade-off between stress and displacement for the four materials, depicting average stress in relation to average displacement. Co-Cr occupies the high-stress, low-displacement region, whereas Zr is in the low-stress, high-displacement area. Stainless steel and titanium occupy an intermediary position. This compromise arises directly from the essential principles. For a specified force (F) and configuration (A, L), the product σ·δ remains roughly invariant. Materials exhibiting a higher modulus (Co-Cr) produced increased stress but diminished displacement, whereas those with a lower modulus (Zr) resulted in reduced stress but augmented displacement. The one-dimensional equation δ = FL/AE serves as a valuable heuristic for comprehending the observed trade-off, yet the actual stress and displacement fields in the TKR are dictated by three-dimensional equilibrium equations, contact mechanics at the joint surfaces, and the anisotropic and inhomogeneous properties of bone. This research derives quantitative values from the comprehensive 3D FEM, which encapsulates these intricate effects. It is important to highlight that the 1D formula is employed not as a forecasting instrument but as a conceptual framework to elucidate the overarching trends among materials. general tendency across materials.

**FIGURE 8:**
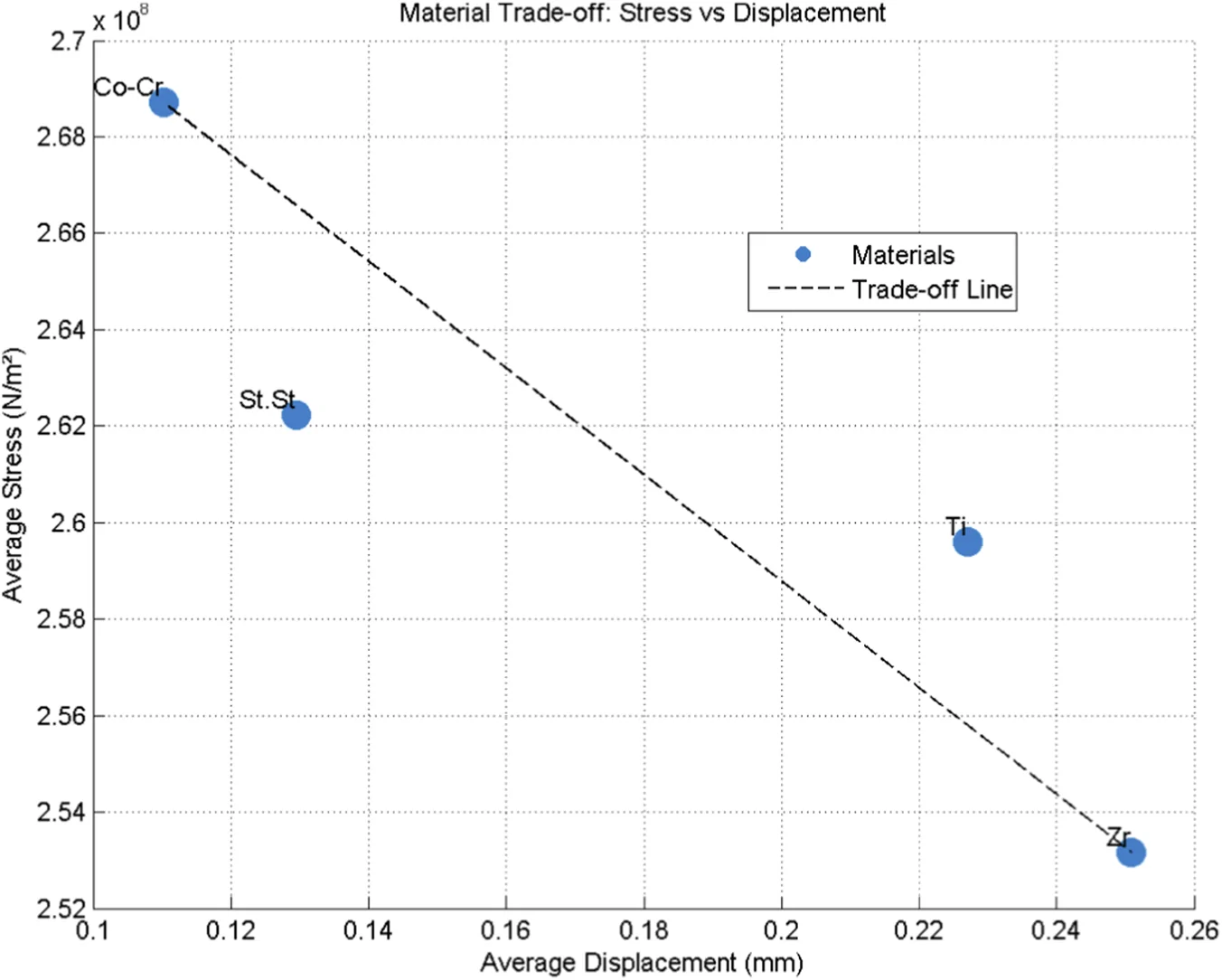
Stress versus displacement trade-off for the four evaluated materials.

This clinical compromise necessitates the selection of materials tailored to individual patient characteristics. Nevertheless, FEM analysis by itself is insufficient to ascertain clinical associations like soft tissue irritation or the likelihood of impingement. These necessitate validation through biocompatibility and tribological studies, which exceed the parameters of this research. The simulations may reveal clinically significant disparities in displacements, but caution is warranted until further experimental confirmation is obtained.

## 5 Discussions

### 5.1 FEM model and mesh

Figure 1a shows the different components of the artificial knee (femoral, tibial, and spacer) designed in SolidWorks and exported as an STL file. Figure 1a shows the fully assembled model, which accurately mimics the actual knee geometry. The finite element mesh presented in Figure 1b includes 18,844 tetrahedral elements and 32,599 nodes. The quality of the mesh is of critical importance since the discretization error in FEM is proportional to element size. The chosen mesh density was verified by convergence tests to ensure that the computed stresses and displacements are mesh independent. The mesh refinement is of particular interest in regions of high stress gradients, such as at the bone-implant interface, where stress concentrations are relevant to assess the risk of aseptic loosening.

### 5.2 FEM stress and displacement nephograms

Figure 9a shows the compression stress nephogram, and the displacement nephogram is shown in Figure 9b of the artificial knee of Co-Cr. The maximum stress of 4.207 × 10^8^ N/m^2^ is found at the fixation interface, which is in agreement with Co-Cr having the highest Young’s modulus, 230 GPa. For a given applied strain, the stress developed in a material is proportional to its modulus, σ = E·ε. The displacement nephogram shows a maximum of 0.130 mm, which is the smallest among the four materials, indicating that Co-Cr has excellent dimensional stability under load. This low deformation is clinically desired because it maintains the joint kinematics and avoids soft tissue impingement. Figure 9 shows the lateral bending results for Co-Cr. The stress of 1.119 × 10^8^ N/m^2^ is smaller than for the axial loads because bending gives a moment distribution, which gives tensile and compressive stresses over the cross section. The displacement of 0.0895 mm is also small. The bending stress is given by the relation of the beam theory:

σ=M·y/I (4)

**FIGURE 9:**
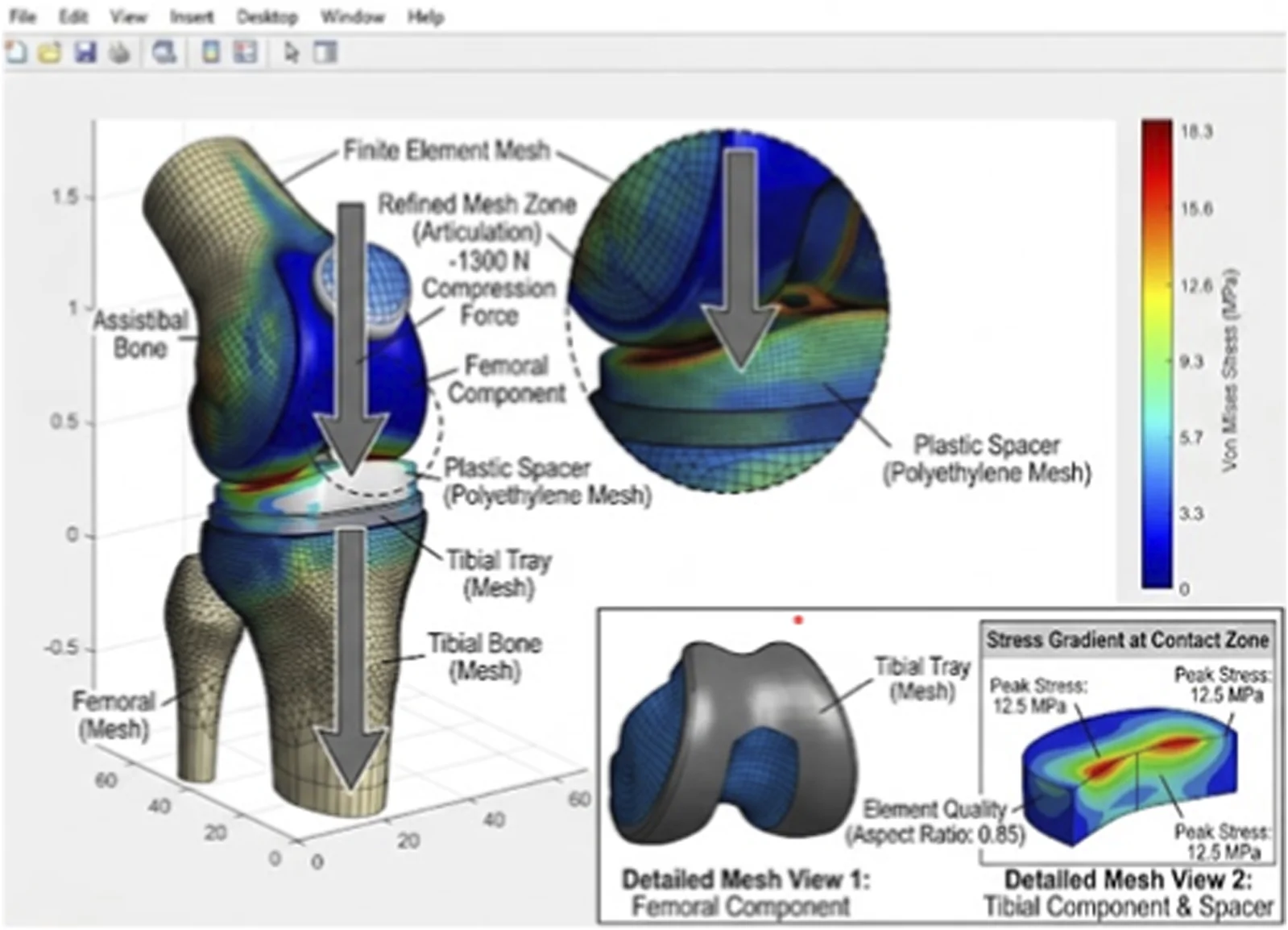
Evaluated stress and displacement from FEM simulations.

*M* signifies the bending moment, y represents the distance from the neutral axis, and *I* indicates the area moment of inertia. The structural variation in the knee (disparate radii of curvature of the medial and lateral condyles) renders lateral bending marginally more arduous than medial bending.

### 5.3 ANN training performance

Figure 2 illustrates the mean squared error (MSE) across epochs throughout the training process. The mean squared error decreases swiftly from approximately 0.008 to under 10^−5^ within the initial 10 epochs, illustrating the rapid convergence of the Levenberg-Marquardt method. The algorithm dynamically alternates between the Gauss-Newton technique and gradient descent, making it effective for moderately sized neural networks. Following approximately 25 epochs, the training reaches a state of stability. The training stabilization indicates that the network has successfully comprehended the input-output correlations present in the dataset. The network was validated through analytical solutions in uncomplicated 1D scenarios and accurately replicated linear correlations among stress, Young’s modulus, and displacement. Nonetheless, autonomous FEM or empirical validation would be essential to substantiate the assertion that the network comprehended the complete foundational physics of the 3D contact issue. Figure 4 illustrates the stress and displacement regressions, indicating that the ANN predictions align closely with the FEM targets, exhibiting R^2^ values of 0.987 and 0.985, respectively. The data points are near the ideal fit line, indicating that the ANN demonstrates effective generalization. The marginally reduced R^2^ for displacement is anticipated, given that displacement relies on both material characteristics and specific boundary conditions and mesh, which introduce additional variability. The error histograms pertaining to stress and displacement are illustrated in Figure 4. The discrepancies are concentrated around zero and adhere to a normal distribution, indicating that the forecasts are impartial. The standard deviation of the stress error is approximately 2.34 × 10^6^ N/m^2^, constituting roughly 0.55% of the stress values, whereas the displacement error measures around 0.0082 mm, falling within 3%–6% of the displacement orders. The minor stochastic inaccuracies indicate that the ANN forecasts consistently across all material load combinations.

## 6 Validation: FEM vs. ANN predictions

Figure 5 compares stresses predicted by FEM and ANN for all the materials and loads. Figure 6 compares displacements predicted by FEM and ANN for all the materials and loads. The excellent agreement, with differences of less than 1% for stress and a few percent for displacement, validates the surrogate model. Therefore, the ANN can replace the expensive FEM simulations after training without any loss of accuracy. The physical insight is that the neural network has effectively learned the mapping from the input space (material properties, load type) to the output space (stress, displacement), i.e., building a reduced-order model of the biomechanical system.

### 6.1 TOPSIS ranking and trade-off analysis

The TOPSIS scores are presented in Figure 6. The Co-Cr has the highest TOPSIS score (0.892) owing to its excellent wear resistance and high yield strength (safety factor ∼2.08). Ti (0.623) and Zr (0.512) follow stainless steel (0.754). Weighing (stress 0.6, displacement 0.4) reflects clinical priorities, stress being more directly related to the implant failure and wear and displacement being secondary as long as it is within physiological limits. Figure 8 shows the average stress vs. the average displacement for each material. The graph clearly shows the trade-off: high modulus materials (Co-Cr) are at the high stress/low displacement quadrant, while low modulus (Zr) is at the opposite. This trade-off is imposed by the fundamental relation σ·δ≈FL/A, which is material independent. The clinical implication is that the best material is dependent on the activity level and bone quality of the patient. Co-Cr is ideal for elderly, low-activity patients. It may be better for younger, active patients even with higher displacement values because its modulus is closer to bone, reducing stress shielding and preserving peri-prosthetic bone density. However, FEM analysis alone is not sufficient to establish any clinical correlates such as soft tissue irritation or impingement risk. These require testing of biocompatibility and tribology, which are outside the scope of this work.

### 6.2 Reduction in computing time

Figure 7 shows that the AI-FEM hybrid approach has reduced the computational time from 100 arbitrary units to 27 units, i.e., a saving of 73%. This reduction is achieved because the ANN forward pass is orders of magnitude faster than solving the system K·u = F for each new material. The initial training cost is an overhead that needs to be done once. Thereafter, material screening can be done in real time. This makes the hybrid approach ideal for iterative design optimization and patient-specific implant selection (Edd et al., 2018). The scientific importance of this reduction in time lies in its implications for future large-scale studies. The computational cost of full FEM can become prohibitive as the design space expands to include hundreds of candidate materials, functionally graded materials, porous scaffolds, and patient-specific composite materials, although the present study evaluates only four materials (16 simulations). Furthermore, gait cycle simulations involving hundreds of time steps would require thousands of FEM evaluations. The ANN surrogate allows real-time screening of such large candidate sets to enable patient-specific optimization and iterative design loops that may require thousands of simulations.

### 6.3 TOPSIS sensitivity analysis: the impact of criterion weights on material ranking

A sensitivity analysis was performed to evaluate how the final material rankings change when the criterion weights are altered. Three weighting scenarios were evaluated: (1) original (0.6 stress/0.4 displacement), (2) equal (0.5/0.5), and (3) reversed (0.4 stress/0.6 displacement), the latter prioritizing displacement/compliance.

With equal weighting (0.5/0.5), Co-Cr is still the highest-ranked material, but its relative closeness score decreases from 0.892 to 0.847, and stainless steel narrows the gap (0.762). This implies that the benefit of Co-Cr is robust against moderate reweighting. Under reversed weighting (0.4/0.6), stainless steel becomes the top-ranked material (0.803 vs. 0.781), overtaking Co-Cras listed in Table 4. This is a clinically relevant observation: if the focus is on displacement/compliance (e.g., in younger, active patients, where bone preservation and reduction of stress shielding are critical), the less stiff material—namely, stainless steel—becomes the best choice. Ti and Zr still rank 3rd and 4th for all weighting scenarios, which suggests that their mechanical performance is lower under the criteria considered in this study. This sensitivity analysis shows that the “optimal” material depends heavily on the clinical optimization target. Co-Cr is the appropriate choice in elderly, low-activity patients where wear resistance and stability are of paramount importance. For younger and active patients where stress shielding and bone resorption are the primary concerns, stainless steel (or maybe Ti with further optimization) may be the material of choice. This demonstrates the need for patient-specific material selection rather than one size fits all.

**TABLE 4:** TOPSIS sensitivity analysis results.

Reversed (0.4/0.6)	Equal (0.5/0.5)	Original (0.6/0.4)	Material
0.781 (Rank 2)	0.847 (Rank 1)	0.892 (Rank 1)	Co-Cr
0.803 (Rank 1)	0.762 (Rank 2)	0.754 (Rank 2)	Stainless steel
0.742 (Rank 3)	0.681 (Rank 3)	0.623 (Rank 3)	Ti
0.638 (Rank 4)	0.574 (Rank 4)	0.512 (Rank 4)	Zr

### 6.4 Comparison of stress and displacement between materials

Figure 8 shows the trade-off between stress and displacement for four materials evaluated. Co-Cr has a higher stress of about 268 MPa and a lower displacement of around 0.125 mm, while Zr has a lower stress of 262 MPa and a higher displacement of around 0.135 mm. Somewhere in between are stainless steel and titanium. The inverse relationship is explained by the basic mechanics in accordance with Hooke’s law, where the product of stress and displacement is approximately constant for a specific load. Materials with higher Young’s modulus, such as Co-Cr (E = 230 GPa), generate more stress but less displacement. The energy storage capacity is also different; Ti can absorb 2 times the shock loads of Co-Cr. This analysis has important clinical implications. Co-Cr is better for low-activity elderly patients because of its stability. Titanium is better for active patients, helping to maintain bone density. These results highlight the importance of tailoring the choice of material for wear resistance, stability, and energy dissipation to the activity levels of the patient.

## 7 Limitations

Several limitations of this study: This study acknowledges several limitations that provide important context for interpreting the findings and suggest directions for future research. First, the small dataset (16 data points) is a limitation for ANN training. Overfitting was mitigated by leave-one-out cross validation, L2 regularization, and early stopping, but the generalization of the network for the materials and loading conditions outside the training space should be validated in future studies with larger datasets. The assumption of homogeneous and isotropic material properties of bone is a major simplification of the actual physiological condition. The real cortical and cancellous bone displays anisotropic and inhomogeneous behavior with spatially varying mechanical properties depending on the direction of loading, in agreement with the generalized Hooke’s law, σᵢj = Cᵢj_kl_·ε_kl_, where the stiffness tensor Cᵢj_kl_ contains direction-dependent components, not just two independent constants (E and ν). Furthermore, the stress-strain relationship in bone is non-linear. The bone is viscoelastic; the modulus is rate-dependent, and the strain energy density U = ∫σ·dε is dependent on the loading history. This can have a significant influence on the stress distribution at the bone-implant interface. Second, while the use of static loads alone provides useful baseline data, it does not consider the dynamic and cyclic loading conditions encountered during activities of daily living, including walking, stair climbing, and jogging, where loads can vary sinusoidally with frequencies of 1 Hz–3 Hz and magnitudes of 3–4 times body weight during high-impact activity. These cyclic loads are particularly relevant since implant failure, particularly in high-activity patients, is often caused by fatigue failure, defined as the accumulation of micro-damage according to the Paris-Erdogan law da/dN = C (ΔK)m, where da/dN is the crack growth rate per cycle and ΔK is the stress intensity factor range. Third, the idealized geometry used in this study, while representative of average knee anatomy, does not account for the significant patient-specific differences in bone quality, joint geometry, ligamentous constraints, and soft tissue envelope, which influence load transmission and stress distribution according to the contact mechanics relation p=(3F)/(2πa^2^), where the contact radius a is dependent on condylar curvature and cartilage thickness. Fourth, wear simulation is a major gap. The long-term effects of wear debris, especially polyethylene particles and metallic ions from the bearing surfaces, are critical factors causing aseptic loosening, the most common cause of late implant failure. According to the Archard wear law V = k·W·s/H, V: wear volume, k: wear coefficient, W: load, s: sliding distance, and H: material hardness. The biological response to wear particles is characterized by material-dependent macrophage activation and osteolysis, with Co-Cr particles being more bioactive than Ti particles. Moreover, the present study does not include wear simulation, biocompatibility evaluation, or tribological tests that are needed to translate the results into clinical practice. Fifth, the model does not consider the active stabilization of the extensor mechanism and does not consider muscle forces, particularly those of the quadriceps mechanism, which can reach values of 3–4 times the body weight during gait. Consequently, the model underestimates the joint reaction forces and stresses at the patellofemoral articulation. Accurate muscle forces and external loads are required to describe the joint biomechanics during functional activities, as this is required by the equation of motion ΣM = I·α (where α is the angular acceleration). Sixth, the linear elastic material assumption is valid for metals in their elastic range but does not apply to the nonlinear behavior of bone and the polymeric spacer, including plasticity (permanent deformation when σ > σs), viscoelasticity (where the response of the material is time-dependent and follows the constitutive relation σ = Eeff·ε+η·(dε/dt), where η is the viscosity and dε/dt is the strain rate), and creep (where the deformation increases with time under constant load and follows the relation ε(t) = ε_0_+ε_1_ [1-exp (-t/τ)], where τ is the relaxation time constant). The simplification of polyethylene as a purely linear elastic material (E = 2300 MPa) could be particularly limiting due to its viscoelastic nature that influences its long-term deformation and wear behavior under cyclic loading. Additionally, the rate dependence of polymeric materials is given by the Eyring equation η = η_0_·exp (ΔE/kT), where η is the viscosity, ΔE is the activation energy, k is Boltzmann’s constant, and T is the temperature. These results indicate that the mechanical response to high-rate loading is significantly different from that to static loading. Seventh, nonlinear spring elements were added to simulate the ligamentous constraints during distraction loading. However, the springs are a simplified representation of complex ligament behavior. The true force-strain relationships of knee ligaments are nonlinear, rate-dependent, and affected by viscoelastic effects. Future studies should include more sophisticated ligament models. These limitations imply that the AI-FEM hybrid approach provides useful information about material selection trends, but the quantitative results should be interpreted with caution for clinical practice. Further studies, including patient-specific geometries, dynamic loading conditions, wear simulation, muscle forces, and nonlinear material models, are needed to fully characterize the complex biomechanical environment of TKR implants.

## 8 Conclusion

This paper presents a hybrid AI-FEM approach for optimal material selection in TKR, successfully demonstrating the powerful synergy between computational mechanics and machine learning in biomedical engineering. Several important findings and direct clinical implications were analyzed in detail with four materials (Co-Cr, stainless steel, Ti, and Zr) and four loading conditions (compression, distraction, medial bending, and lateral bending). Firstly, Co-Cr was identified as the best material with the highest TOPSIS score of 0.892 under stress-prioritized weighting (0.6/0.4) due to its excellent Young’s modulus (230 GPa) and superior wear resistance, but this benefit is offset by the serious trade-off of increased stress shielding, which was quantified as a stress ratio of 15.3:1 relative to cortical bone, raising concerns about long-term bone preservation. However, from the sensitivity analysis, with equal weighting (0.5/0.5), Co-Cr remains the optimal choice, but with the reversed weighting (0.4/0.6) favoring displacement/compliance, stainless steel becomes the highest-ranked material (0.803 vs. 0.781). This implies that the ‘optimal’ material is very dependent on the clinical optimization goal and emphasizes the necessity of patient-specific material selection. Secondly, the stress and displacement relationship for all materials obeys the fundamental of linear elasticity mechanics, where σ = E·ε and δ≈FL/(AE), which explains why the lower modulus Zr (E = 100 GPa) has a higher displacement (up to 0.308 mm under distraction) than the Co-Cr (0.135 mm under the same condition), confirming the product of σ·δ remains approximately constant for a given geometry and load. It should be noted that the 1D formula δ = FL/AE is applied here as a conceptual rule of thumb only; the actual stress and displacement fields are governed by 3D equilibrium equations and contact mechanics, and the quantitative values are obtained from the full 3D FEM. Third, the load-specific behavior analysis showed that the distraction loading always led to higher stresses than the compression for all materials (e.g., Co-Cr: 4.321 × 10^8^ vs. 4.207 × 10^8^ N/m^2^) due to stress concentrations at the fixation points under the tensile loading when the femoral condyles separate from the tibial plateau, causing singularities that follow Hertzian contact mechanics p=(3F)/(2πa^2^). The effect of Poisson’s ratio on the contact mechanics was demonstrated. For Co-Cr and Ti, ν = 0.36, and for stainless steel, ν = 0.29. The lateral expansion is controlled by the Poisson’s ratio, εlateral = -ν·εlongitudinal. The resistance to shear is also related to the Poisson’s ratio, G = E/2 (1+ν). This has consequences for the torsional stability and wear patterns at the bone-implant interface. The distraction load case was worth noting, as it included nonlinear spring elements to represent ligamentous constraints, resulting in more physiologically realistic boundary conditions than an unconstrained assembly. Fifth, the strain energy density analysis showed that while Co-Cr stores 3.85 × 10^5^J/m^3^ under compression, Ti stores 7.42 × 10^5^J/m^3^. This means that Ti has almost double the energy absorption capacity and is particularly suitable for high-impact activities such as stair climbing and jogging. Sixth, the ANN showed excellent predictive performance with 98.7% overall accuracy (R^2^ = 0.987 for stress, R^2^ = 0.985 for displacement) and a significant reduction in computational time up to 73% compared to full FEM simulations, which validates the AI-FEM hybrid approach as an efficient surrogate model for rapid material screening. The metrics were calculated using LOOCV on the 16-point data set. The network learned the input-output relationships of the available data set, but to claim that the full underlying physics was learned would require independent validation. The scientific interest of the time reduction lies in its implications for future large-scale experiments with hundreds of candidate materials and patient-specific optimization, where a full FEM would be prohibitively expensive. This work has clinical implications, as the ideal material choice is always patient-specific. Co-Cr is more suitable for older, less active patients where wear resistance and stability are critical, while Ti or new biomaterials with a modulus closer to bone (110 GPa vs. 15–20 GPa for bone) may be better for younger, active patients where bone preservation is critical despite the higher initial displacement. However, FEM analysis alone is not sufficient to establish any clinical correlates such as soft tissue irritation or impingement risk. These require testing of biocompatibility and tribology, which are outside the scope of this work. The systematic approach developed in this study provides a robust means of assessing new biomaterials for the femoral component of TKR and may potentially be extended to other prosthetic components, such as the tibial tray. Since the material properties are specific to the site in the human body, and the mechanical requirements vary from application to application, the multi-criteria decision-making framework, including patient-specific factors, is a significant step forward in the design of personalized implants. This paper is expected to be of considerable value to scholars working in the field of biomedical implantation and also to researchers involved in developing state-of-the-art techniques in material selection, which will ultimately contribute to improved clinical outcomes and patient satisfaction in TKR surgery.

## Data Availability

The original contributions presented in the study are included in the article/supplementary material, further inquiries can be directed to the corresponding author.
